# Analysis of Suicide Methods and Substances Influencing the State of Consciousness of Their Victims in Poland

**DOI:** 10.3390/ijerph18094936

**Published:** 2021-05-06

**Authors:** Dorota Lasota, Dagmara Mirowska-Guzel, Krzysztof Goniewicz

**Affiliations:** 1 Department of Experimental and Clinical Pharmacology, Medical University of Warsaw, 02097 Warsaw, Poland; dlasota@wum.edu.pl; 2 Department of Aviation Security, Military University of Aviation, 08521 Dęblin, Poland; k.goniewicz@law.mil.pl

**Keywords:** suicide, suicide attempts, methods of suicide, alcohol, presuicidal prophylaxis, psychoactive substances

## Abstract

An integral part of the World Health Organization’s (WHO) mental health action plan for 2013–2020 is suicide prevention, and a 10% reduction in the suicide rate. A key element of the preventive measures taken is, among other things, limiting access to means enabling and facilitating committing suicide. However, this requires detailed knowledge of community suicide patterns and preferences. Unfortunately, such information is not usually collected, and the recorded method of committing suicide is often imprecise and untrue, which means that such knowledge has limited application in presuicidal prophylaxis. The statistical data on suicides recorded as part of the Police Statistics in Poland during the years 2009–2019 was analyzed. The analysis included suicide deaths and suicide attempts, taking into account the suicide methods and substances influencing the state of consciousness of their victims. The number of suicides in Poland in the analyzed period tended to increase. The most common method of suicide was hanging, and alcohol was the substance influencing the state of consciousness of suicide victims. The statistics for 2017–2019 showed the presence of new methods of suicide, such as taking drugs other than hypnotics, intoxication with narcotic drugs, poisoning with designer drugs, suffocation and self-immolation, as well as new psychoactive substances affecting the state of consciousness of victims, i.e., drugs and narcotic drugs. The effectiveness of actions taken in Poland in the field of suicide prevention does not bring the desired effects, as evidenced by growing number of suicides. This low effectiveness is mainly due to the lack of a national suicide prevention strategy. The effectiveness of these activities may be improved by creating a uniform database of suicides, which is a source of reliable information which will allow the taking of actions aimed at limiting the availability of means enabling and facilitating the committing of suicide. The study aimed to analyze the types of suicide methods and substances influencing the state of consciousness of their victims in Poland in the years covered by the study.

## 1. Introduction

Suicide is an act of deliberately taking one’s own life. It results from a complex interaction of risk factors such as age, gender, marital status, socioeconomic status, social factors, physical condition, mental illness or life events and the presence of triggers, including sudden mental disorders and intoxication with alcohol or other psychoactive substances. [1]. As a result of suicide, death occurs in 7% to 18% of people addicted to alcohol [2,3], with 14–43% attempting suicide [4,5,6]. Within this group, men are four times more likely than women [7] to be prone to attempt suicide [8]. They are also more likely people who are suffering from somatic diseases, rather than being those without such burdens [9]. Moreover, similarly to the general population, also in this group, the coexistence of other mental disorders increases the risk of attempting suicide. This coexistence mainly concerns affective diseases [10,11], but also sleep disorders [7] and addiction to other psychoactive substances, including, for example, nicotine [12].

Suicide is a global phenomenon which impacts all countries. Up to 75% of suicides occur in low and middle-income countries (LMICs) [13]. Poland is one of the leaders among the countries with a high suicide risk, both in Europe and globally (the suicide rate per 100,000 people for 2016 was 16.2). The scale of this phenomenon is evidenced by the fact that in 2019 more people died of suicide in Poland than in road accidents (*n* = 5255 vs. *n* = 2909, respectively) [14].

A key element of the suicide reduction effort is, among other things, restricting access to means of enabling and facilitating suicide. However, this requires detailed knowledge of community suicide patterns and preferences. Unfortunately, such information is not usually collected, and the described suicide methods are often imprecise and untrue, which means that such data are of limited use in pre-suicidal prophylaxis.

The study aimed to analyze the types of suicide methods and substances influencing the state of consciousness of their victims in Poland in the years covered by the study.

## 2. Materials and Methods

The authors analyzed the statistical data on suicides recorded as part of Police Statistics in Poland in 2009–2019. The analysis included suicide deaths and suicide attempts, taking into account the suicide methods and substances influencing the state of consciousness of their victims. The analysis did not include 70 suicide attempts recorded in 2013 due to the lack of detailed, analyzed data. The results were gathered by compiling and comparing the collected data using Microsoft Office Excel (Microsoft, Redmond, WA, USA).

## 3. Results

**Figure 1:**
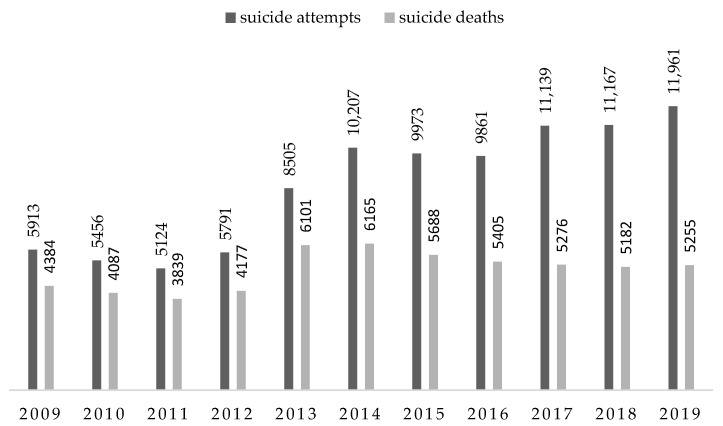
Number of suicide deaths and suicide attempts victims in Poland in 2009–2019. Source: own study based on https://statystyka.policja.pl/st/wybrane-statkieta/zamachy-samobojcze (accessed on 30 November 2020).

The number of suicides in Poland in the analyzed period tended to increase. There were *n* = 95,097 suicide attempts. Most in 2019, *n* = 11,961 and the lowest in 2011, *n* = 5124. Suicide deaths accounted for over 58.42% (*n* = 55,559) of all events. Most in 2014, *n* = 6165 and the lowest in 2011, *n* = 3839 (Figure 1).

The most frequently used method of suicide attempts in the analysed period in Poland was hanging, *n* = 57,740 (60.72%). The highest number of victims was recorded in 2010, *n* = 3973 (72.82%), and the lowest in 2019, *n* = 5740 (47.99%). It has been observed that since 2011 the percentage of suicide attempts committed with this method has been decreasing. The second most frequent method was jumping from a height, *n* = 7462 (7.85%), and the next most frequent method was self-harm, *n* = 6853 (7.20%). Attention is drawn to the significant percentage of suicide attempts for which the method has not been specified, *n* = 5684 (5.98%).

In the statistics for 2017–2019, the presence of new methods was noted, such as taking drugs other than hypnotics, intoxication with narcotic drugs, poisoning with designer drugs, suffocation and self-immolation (Table 1).

The highest number of suicide deaths was recorded in 2014, and the lowest in 2011 [respectively: *n* = 6165 (11.09%), *n* = 3839, (6.91%)]. The leading method was hanging, *n* = 46 381, (83.48%). The highest number of victims was recorded in 2010, *n* = 3518, (86.08%), and the lowest in 2019, *n* = 4240, (80.68%). Since 2015, the percentage of suicides committed by hanging has been declining. The next most frequently used methods were jumping from a height and throwing oneself under a moving vehicle [*n* = 3296 (5.93%), *n* = 985, (1.77%), respectively].

In the statistics for 2017–2019, the presence of new methods was noted, such as taking drugs other than hypnotics, intoxication with narcotic drugs, poisoning with designer drugs, suffocation and self-immolation (Table 2).

**Figure 2:**
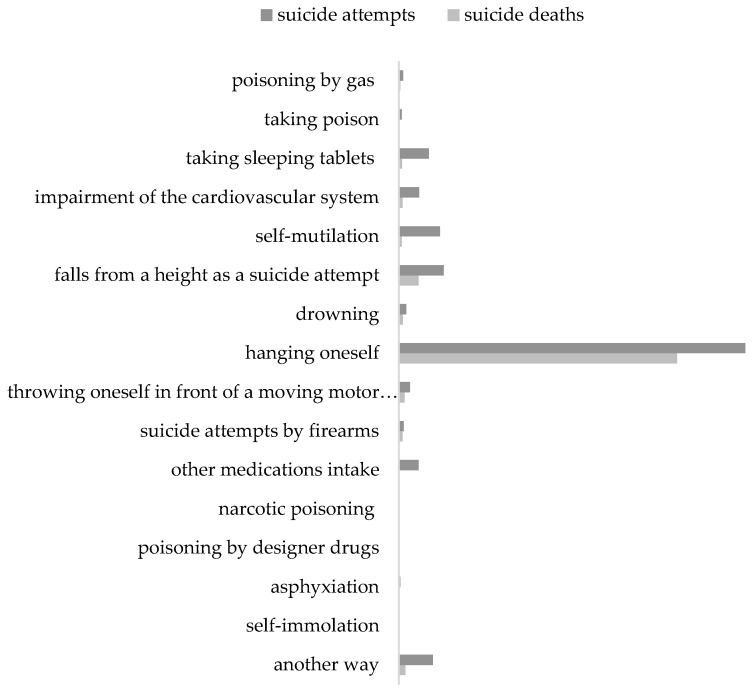
Methods of suicide deaths and suicide attempts in Poland, 2009–2019. Source: own study based on https://statystyka.policja.pl/st/wybrane-statkieta/zamachy-samobojcze (accessed on 30 November 2020).

The list of suicide methods in the analyzed period in Poland, broken down into suicide deaths and suicide attempts, is presented in Figure 2.

The analysis showed that the most frequently used substance influencing the state of consciousness of victims of suicide attempts was alcohol, *n* = 26,894 (27.94%). The highest number of victims was recorded in 2017, *n* = 3635 (32.63%), and the lowest in 2013, *n* = 1857 (21.83%). It has been observed that since 2018, the percentage of suicide attempts committed under the influence of alcohol has been declining. Medicines were the second most frequently used substance, *n* = 2470 (2.57%). 0.65% (*n* = 631) of the victims were under the influence of the designer drugs. For nearly 58% (*n* = 55,407) of victims, the state of consciousness was not established (Table 3).

The statistics for 2017–2019 noted the presence of new items describing the sobriety of victims, taking into account the impact of new psychoactive substances such as narcotic drugs and drugs on this state (Table 3).

**Figure 3:**
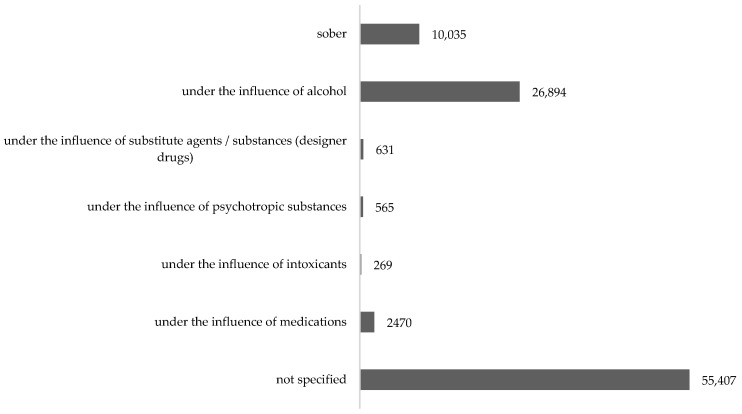
State of awareness of the victims of suicide attempts in Poland, 2009–2019. Source: own study based on https://statystyka.policja.pl/st/wybrane-statkieta/zamachy-samobojcze (accessed on 30 November 2020).

The state of consciousness of victims of suicide attempts in Poland in the analyzed period, taking into account individual psychoactive substances influencing this state, is presented in Figure 3.

## 4. Discussion

Suicide is an act of deliberately taking one’s own life, and a suicide attempt is any non-fatal suicidal behavior which may have been committed with or without intent to take one’s own life.

Suicidal behavior, and especially the preferred method of suicide, varies by country. According to the WHO, the most common ways of committing suicide are poisoning with pesticides, hanging and jumping from a height. The deliberate ingestion of pesticides is one of the key methods of suicide in LMICs. It mainly concerns rural areas and agricultural regions of the world. Hanging accounts for 50% of suicides in highly developed countries, and 18% are caused by the use of firearms. A relatively high percentage of suicides with firearms occurs in highly developed countries in America (especially in countries where firearms are common in private households), where it is the cause of 46% of all suicides. In other highly developed countries, suicides by firearms account for only 4.5%. In heavily urbanized areas such as China, Hong Kong SAR and Singapore, jumping from a height, i.e., from residential buildings, is common. In Hong Kong in 1998, the “epidemic” of grilling charcoal to produce highly toxic carbon monoxide as a means of committing suicide began and spread rapidly to Taiwan and China. There it has become the most popular method of committing suicide in recent years. In other parts of the world, hydrogen sulfide is used for suicide purposes (including in Japan), as well as helium [13]. Unfortunately, data on suicides for entire regions or individual countries does not consider the variability of suicide rates, demographic patterns and methods used within each country [15,16].

In Poland, the most common method of suicide is hanging, mainly among men, while women most often try to take large amounts of drugs [17,18,19,20,21]. Additionally, in the presented analysis, hanging was the leading method of suicide. In general, in the case of suicide deaths, more radical methods which guarantee certain death, such as the aforementioned hanging, jumping from a height, throwing oneself under a moving vehicle (most often a train), drowning or shooting oneself, prevailed [19,20,22]. Hanging, jumping from a height, self-harm, impairment of the cardiovascular system or taking sleeping pills (and other medications) are the most common methods of suicide attempts (Figure 2).

Changing patterns also apply to substances which impact upon the state of consciousness of suicide victims. Consciousness disorders caused by alcohol consumption or the use of other psychoactive substances are found in 25–50% of all suicide victims [23]. Drinking alcohol can be attributed to 22% of all suicides, which would seem to demonstrate that one in five suicides would not have occurred if the population had not consumed alcohol [24]. In long-term drinkers, the chance of frequent intoxication increases, and thus the risk of suicide increases [25,26,27,28]. Nevertheless, even a single consumption of alcohol may be a risk factor for suicide, as confirmed by the American data on the presence of alcohol in the blood of 70% of people attempting suicide, and up to 66% of suicide deaths [29]. 

Alcohol helps to reduce the threshold of aggression, the level of control of emotions and behaviour, self-esteem, the concentration of attention and the ability to assess the situation and the emergence of suicidal thoughts adequately. There is a correlation between the average amount of alcohol consumed in the general population and the number of suicides. The results of numerous studies have shown that with the increase in the average amount of alcohol drunk per capita, an increase in the number of suicides was observed [30,31,32,33,34]. However, there is no simple linear relationship here because the drinking model and its social context also play an essential role in shaping the risk of suicide [35,36]. In Southern European countries, where alcohol is consumed frequently but in small amounts, the risk of suicide is lower than in Scandinavian countries, where alcohol is consumed in large amounts at a time, while significantly increasing the risk of suicidal behavior [36,37]. The gender of the victims is also important. In Polish studies, blood alcohol at the time of suicide was found in 31% of women and 43% of men. In Finland—19% of women [37,38], in Slovenia—18% [39] and in Scotland, the presence of alcohol in blood was found in 37% of women [40,41].

Combining alcohol with other psychoactive substances intensifies its effects and may have tragic consequences, especially for people with mental problems. These substances include medications, among others. The most common are benzodiazepine sedatives and hypnotics, followed by antiepileptic drugs (carbamazepine, valproic acid), antidepressants (tricyclics, serotonin reuptake inhibitors) and neuroleptics (classic and atypical). This problem largely impacts psychiatric patients, people addicted to medications (mostly benzodiazepines) and alcohol addicts (people addicted to alcohol use psychotropic drugs to reduce discomfort at the onset of withdrawal symptoms or symptoms of acute alcohol intoxication). Thus, there may be mixed poisoning, multi-drug or alcohol-drug poisoning [42]. 

Psychoactive substances are also many over the counter (OTC) drugs, those which contain substances which can alter consciousness, behavior and feeling when consumed in large amounts. Among the OCT drugs, the most popular are dextromethorphan (a synthetic analog of codeine), as well as ephedrine and pseudoephedrine [43].

In, addiction to psychoactive substances other than alcohol, incl. cannabinol, heroin or nicotine can cause the appearance or worsening of symptoms of depression, which may be accompanied by suicidal thoughts and attempts to implement them [24,44]. In the research by Nowicka et al. on suicide attacks of people under the influence of psychoactive substances in the years 1990–2015 (excluding suicides after taking poisons), it was found that 58% of victims were under the influence of ethanol, and in 4% the presence of psychoactive substances other than ethanol was found, including volatile organic solvents, amphetamines, opiates, barbiturates, cannabinoids as well as jointly taken: opiates and benzodiazepines, opiates and barbiturates, opiates and cannabinols, opiates, benzodiazepines and barbiturates (the study omitted suicides after taking new psychoactive substances, the so-called intoxicants). They were often victims of hangings, falls from heights, gunshots, cuts and traffic accidents, mainly railway accidents [45].

One of the new psychoactive substances (NPS) present in the statistics of the Polish Police is designer drugs. Those currently used are drug substitutes or mixtures of drugs. Thanks to the potentials of chemical synthesis, their diversity is practically unlimited. They may contain unknown chemical compounds, the effects of which are difficult to predict. They contain psychoactive compounds such as N-benzylpiperazine (amphetamine substitute), synthetic cannabinoids, cathinone derivatives (mephedrone, naphyrone) with structural similarity to ephedrine cathine and other amphetamine derivatives. Their concentration is often much higher than in traditional illegal drugs, which can result in their overdose or even death [46]. In the studies by Zawilska et al., mephedrone was identified in postmortem examinations in 90 cases, 18 of which were suicides, including 11 by hanging. The sole use of mephedrone resulted in 8 deaths [47]. In the studies by Skowronek et al. in terms of the presence of NPS in the biological material secured during an autopsy in 2013–2015, suicides of young people (16–40 years old) under the influence of substances are classified as NPS were revealed. The most common methods of suicide in these cases were hanging, falling from a height, cutting wounds and bleeding out. The most popular group of NPS adopted by the above-mentioned victims were synthetic cathinone and synthetic cannabinoids. The presence of ethyl alcohol in the blood was found in 2 cases [48].

The obtained results show that the vast majority of victims of suicide attempts for whom the state of consciousness was established was under the influence of psychoactive substances. Most of them were victims under the influence of alcohol (27.94%). Detailed statistics of suicide attempts in the years 2017–2019 indicate a growing percentage of victims under the influence of drugs (Figure 3). The reasons for this situation can be seen, among other things, in the unlimited availability of many drugs. Therefore, despite the known the method of committing suicide, it seems justified to establish the circumstances accompanying suicide actions, as extremely important for preventive measures taken for individual risk factors, which undoubtedly include alcohol and other psychoactive substances.

The presented analysis results prove the significant role of continuous monitoring of suicides in the face of changing patterns. Meanwhile, data on the methods used are limited, as such information is usually not collected. There are no, or if so only quite basic, systems for collecting data on suicides and suicide attempts in many countries. For example, the United States of America (USA) has such a system. The National Violent Death Reporting System (NVDRS) is a surveillance system which collects detailed statistical data on each case of violent death, including suicides [13,49,50].

There is no national suicide database in Poland. Official suicide statistics come from two databases with different data collection mechanisms. These are the National Police Information System and the Central Statistical Office [51,52]. Until 2012, data on suicides were collected in the National Police Information System after the screening had been conducted and completed. From 2013, data are entered immediately after the incident, i.e., when it is established that a suicide attack took place, and the system allows for their modification if it is determined at a later stage of the proceedings that no suicide attack took place. It is worth mentioning that this change in the data collection strategy was almost immediately reflected in the police statistics, because already in 2013 there was a significant increase in the number of registered suicides, especially suicide attempts (Figure 1). In the case of the Central Statistical Office, data is updated from Death Records, and suicide attempts are not reported at all. Such a situation results in significant discrepancies in the reported data, which do not reflect the actual situation and require the development of an optimal form of their registration, enabling reliable data.

However, regardless of the database, many methods, especially the new ones, are not identified by the commonly used International Classification of Diseases and Health Problems—X Revision (ICD-10), (ICD-10 suicide codes are in the range X60-X849). They are often described as “external cause of death,” which is imprecise and untrue [13,53]. Moreover, it was only in 2018 when the obligation to register suicide attempts on hospital treatment cards, including psychiatric treatment, was introduced. Previously, there was no obligation to report such events, and information about people after suicide attempts under the care of medical facilities was not included in the statistics. Hence, Poland’s actual number of suicides was and is probably higher than the one quoted in the statistics.

The number of countries which launch appropriate suicide prevention programs in their country is growing year by year, bringing tangible results, as the number of suicides worldwide decreased by almost 10% in 2010–2016 [13]. Unfortunately, not in Poland. To achieve the goals, set by the WHO, it is necessary to develop and implement the National Suicide Prevention Program, which is not available in Poland, as in other countries. In some countries, e.g., in Japan, it has the status of law. It is in Japan, among others, where because of suicide prevention measures taken, a decrease by 10% has occurred, while in Chile the number is 15% and in Scotland 18% [54].

In Poland, there is only a Working Team to prevent suicides and depression at the Public Health Council at the Ministry of Health. It was established in 2016 to provide substantive support for implementing the National Health Program 2016–2020 in the field of depression and suicide prevention for the global goal of reducing the suicide rate. In December 2017, at the request of the Ministry of Health, as part of the implementation of the above-mentioned The National Health Program has launched the Support Center for people in a state of mental crisis, where 24-h and free assistance is provided by qualified specialists, i.e., psychologists, lawyers and social workers. 

In addition, there are mobile crisis programs, such as the Crisis Helpline for Children and Youth, or the Crisis Hotline for Adults.

Unfortunately, an increase in the number of suicides in Poland has been still observed. Therefore, it is difficult to talk about the effectiveness of actions taken in this area [55]. In these circumstances, it is of particular importance to increase the knowledge and awareness of the whole society about mental health.

To sum up, given the increasing number of committed suicides in Poland, early identification of areas which should be the subject of intervention is of key importance. Limiting access to means which enable and facilitate committing suicide seems to be a priority here. It is critical to intervene at both national and local levels, in order to restrict access to means of suicide, such as pharmaceuticals and firearms and structural interventions, by restricting access to bridges, buildings and railroads [56,57,58,59,60]. For the effectiveness of these activities, continuous, multi-sectoral cooperation of government administration, local government units, public organizations, communities and the media is necessary. In addition, and perhaps even above all, obligatory and reliable reporting of all suicides and suicide attempts to a uniform database, which should improve surveillance and enable the generation of reliable data.

## 5. Limitations

The analysis was carried out at the national level, so the obtained results do not take into account the local variability in the applied suicide methods and substances influencing the state of consciousness of their victims. Moreover, the lack of statistical data on the state of awareness of victims of suicide deaths by 2017 made it impossible to conduct a full study in this area, i.e., a study assessing the state of awareness of victims broken down into suicide deaths and suicide attempts in the analyzed period.

## 6. Conclusions

The effectiveness of actions taken in Poland in the field of suicide prevention does not bring the desired results, as evidenced by their growing number. This low effectiveness is mainly due to the lack of a national suicide prevention strategy. The effectiveness of these actions may be improved by creating a uniform database of suicides, which will be a source of reliable information which will allow for the undertaking of actions to limit the availability of means enabling and facilitating the committing of suicide.

## Figures and Tables

**Table 1:** Number and percentage of suicide attempts victims depending on the suicide method.

Method	Number and Percentage of Suicide Attempts Victims											General
	2009	2010	2011	2012	2013	2014	2015	2016	2017	2018	2019	
Poisoning by gas/exhaust fumes	41 (0.69)	38 (0.70)	39 (0.76)	33 (0.57)	47 (0.55)	67 (0.66)	85 (0.85)	73 (0.74)	92 (0.83)	104 (0.93)	114 (0.95)	733 (0.77)
Poisoning with chemicals/ toxic substances	30 (0.51)	26 (0.48)	20 (0.39)	30 (0.52)	41 (0.48)	52 (0.51)	52 (0.52)	49 (0.49)	62 (0.56)	68 (0.61)	89 (0.74)	519 (0.55)
Taking sleeping tablets/psychotropic tablets	199 (3.36)	214 (3.92)	192 (3.75)	193 (3.33)	314 (3.69)	474 (4.64)	520 (5.22)	572 (5.80)	674 (6.05)	774 (6.93)	894 (7.48)	5020 (5.28)
Impairment of the cardiovascular system	156 (2.64)	154 (2.82)	155 (3.02)	143 (2.47)	226 (2.66)	370 (3.62)	367 (3.68)	393 (3.99)	388 (3.48)	501 (4.49)	524 (4.38)	3377 (3.55)
Superficial self-mutilation	227 (3.84)	191 (3.50)	179 (3.49)	215 (3.71)	400 (4.70)	652 (6.39)	788 (7.90)	816 (8.28)	991 (8.90)	1095 (9.80)	1299 (10.86)	6853 (7.20)
Falls from a height as a suicide attempt	408 (6.90)	323 (5.92)	325 (6.34)	443 (7.65)	675 (7.94)	856 (8.39)	877 (8.79)	798 (8.09)	934 (8.38)	911 (8.16)	912 (7.63)	7462 (7.85)
Drowning	108 (1.83)	88 (1.61)	100 (1.95)	105 (1.82)	118 (1.39)	113 (1.11)	119 (1.19)	126 (1.28)	127 (1.14)	117 (1.05)	114 (0.95)	1235 (1.29)
Hanging oneself	4265 (72.13)	3973 (72.82)	3706 (72.33)	4059 (70.09)	5955 (70.01)	6582 (64.49)	6066 (60.82)	5819 (59.01)	5966 (53.55)	5609 (50.23)	5740 (47.99)	57,740 (60.72)
Throwing oneself in front of a moving motor vehicle	81 (1.37)	103 (1.89)	77 (1.50)	110 (1.90)	158 (1.86)	184 (1.80)	207 (2.08)	216 (2.19)	241 (2.16)	222 (1.99)	275 (2.30)	1874 (1.97)
Suicide attempts by firearms	46 (0.78)	44 (0.81)	32 (0.63)	58 (1.00)	83 (0.98)	86 (0.84)	79 (0.79)	104 (1.05)	90 (0.81)	96 (0.86)	102 (0.85)	820 (0.86)
Other medications intake	-	-	-	-	-	-	-	-	959 (8.61)	1061 (9.50)	1289 (10.78)	3309 (3.48)
Narcotic poisoning	-	-	-	-	-	-	-	-	21 (0.19)	17 (0.15)	19 (0.16)	57 (0.06)
Poisoning by designer drugs	-	-	-	-	-	-	-	-	3 (0.03)	3 (0.03)	0 (0.00)	6 (0.01)
Asphyxiation	-	-	-	-	-	-	-	-	89 (0.80)	84 (0.75)	93 (0.78)	266 (0.28)
Self-immolation	-	-	-	-	-	-	-	-	48 (0.43)	54 (0.48)	40 (0.33)	142 (0.15)
Another way	352 (5.95)	302 (5.53)	299 (5.84)	402 (6.94)	488 (5.74)	771 (7.55)	813 (8.16)	895 (9.08)	454 (4.08)	451 (4.04)	457 (3.82)	5684 (5.98)
Overall	5913 (6.22)	5456 (5.74)	5124 (5.39)	5791 (6.09)	8505 (8.94)	10,207 (10.73)	9973 (10.49)	9861 (10.37)	11,139 (11.71)	11,167 (11.74)	11,961 (12.58)	95,097 (100.00)

Note: data concerning a victim may appear in several items. Source: own study based on https://statystyka.policja.pl/st/wybrane-statkieta/zamachy-samobojcze (accessed on 30 November 2020).

**Table 2:** Number and percentage of suicide deaths victims depending on the suicide method.

Method	Number and Percentage of Suicide Deaths Victims											General
	2009	2010	2011	2012	2013	2014	2015	2016	2017	2018	2019	
Poisoning by gas/exhaust fumes	11 (0.25)	17 (0.42)	25 (0.65)	13 (0.31)	15 (0.25)	18 (0.30)	37 (0.65)	21 (0.40)	29 (0.55)	38 (0.73)	41 (0.78)	265 (0.48)
Poisoning with chemicals/ toxic substances	15 (0.34)	11 (0.27)	9 (0.24)	13 (0.31)	14 (0.23)	14 (0.23)	19 (0.33)	13 (0.24)	17 (0.32)	12 (0.23)	24 (0.46)	161 (0.29)
Taking sleeping tablets/psychotropic tablets	33 (0.75)	38 (0.93)	41 (1.07)	37 (0.88)	59 (0.97)	69 (1.12)	45 (0.79)	66 (1.22)	44 (0.83)	48 (0.93)	50 (0.95)	530 (0.95)
Impairment of the cardiovascular system	38 (0.87)	39 (0.95)	33 (0.86)	36 (0.86)	56 (0.92)	53 (0.86)	62 (1.10)	51 (0.94)	75 (1.42)	83 (1.60)	96 (1.83)	622 (1.12)
Superficial self-mutilation	40 (0.91)	31 (0.76)	30 (0.78)	30 (0.72)	57 (0.93)	54 (0.88)	69 (1.21)	71 (1.31)	46 (0.87)	36 (0.69)	38 (0.72)	502 (0.90)
Falls from a height as a suicide attempt	231 (5.27)	165 (4.04)	185 (4.82)	243 (5.82)	392 (6.43)	373 (6.05)	361 (6.35)	344 (6.36)	342 (6.48)	330 (6.37)	330 (6.28)	3296 (5.93)
Drowning	80 (1.82)	69 (1.69)	78 (2.03)	78 (1.87)	73 (1.20)	52 (0.84)	51 (0.90)	65 (1.20)	57 (1.08)	46 (0.89)	45 (0.86)	694 (1.25)
Hanging oneself	3726 (85.00)	3518 (86.08)	3274 (85.28)	3495 (83.67)	5142 (84.30)	5241 (85.01)	4748 (83.47)	4473 (82.76)	4313 (81.75)	4211 (81.26)	4240 (80.68)	46 381 (83.48)
Throwing oneself in front of a moving motor vehicle	60 (1.37)	77 (1.88)	50 (1.30)	76 (1.82)	99 (1.62)	91 (1.47)	90 (1.58)	100 (1.85)	105 (1.99)	105 (2.03)	132 (2.51)	985 (1.77)
Suicide attempts by firearms	37 (0.84)	39 (0.95)	30 (0.78)	47 (1.13)	67 (1.10)	63 (1.02)	53 (0.93)	70 (1.30)	72 (1.36)	79 (1.52)	86 (1.64)	643 (1.16)
Other medications intake	-	-	-	-	-	-	-	-	58 (1.10)	61 (1.18)	63 (1.20)	182 (0.33)
Narcotic poisoning	-	-	-	-	-	-	-	-	4 (0.08)	3 (0.06)	4 (0.07)	11 (0.02)
Poisoning by designer drugs	-	-	-	-	-	-	-	-	0 (0.00)	0 (0.00)	0 (0.00)	0 (0.00)
Asphyxiation	-	-	-	-	-	-	-	-	49 (0.93)	45 (0.87)	44 (0.84)	138 (0.25)
Self-immolation	-	-	-	-	-	-	-	-	12 (0.23)	15 (0.29)	11 (0.21)	38 (0.07)
Another way	113 (2.58)	83 (2.03)	84 (2.19)	109 (2.61)	125 (2.05)	137 (2.22)	153 (2.69)	131 (2.42)	53 (1.01)	70 (1.35)	51 (0.97)	1109 (2.00)
Overall	4384 (7.89)	4087 (7.36)	3839 (6.91)	4177 (7.52)	6101 (10.98)	6165 (11.09)	5688 (10.24)	5405 (9.73)	5276 (9.49)	5182 (9.33)	5255 (9.46)	55 559 (100.00)

Note: data concerning a victim may appear in several items. Source: own study based on https://statystyka.policja.pl/st/wybrane-statkieta/zamachy-samobojcze (accessed on 30 November 2020).

**Table 3:** Number and percentage of suicide attempts victims depending on the state of consciousness.

Consciousness Level	Number and Percentage of Suicide Attempts Victims											General
	2009	2010	2011	2012	2013	2014	2015	2016	2017	2018	2019	
Sober	668 (11.29)	592 (10.85)	500 (9.76)	618 (10.67)	683 (8.03)	901 (8.83)	867 (8.69)	987 (10.01)	1306 (11.72)	1342 (12.02)	1571 (13.13)	10 035 (10.42)
Under the influence of alcohol	1453 (24.57)	1341 (24.58)	1258 (24.55)	1438 (24.83)	1857 (21.83)	2734 (26.78)	2841 (28.48)	2899 (29.39)	3635 (32.63)	3634 (32.54)	3804 (31.80)	26 894 (27.94)
Under the influence of substitute agents/substances (designer drugs)	44 (0.74)	47 (0.86)	31 (0.60)	47 (0.81)	66 (0.78)	106 (1.04)	118 (1.18)	98 (0.99)	31 (0.28)	21 (0.19)	22 (0.18)	631 (0.65)
Under the influence of psychotropic substances	44 (0.74)	32 (0.59)	27 (0.53)	45 (0.78)	62 (0.73)	109 (1.07)	133 (1.33)	113 (1.15)	-	-	-	565 (0.59)
Under the influence of intoxicants	-	-	-	-	-	-	-	-	79 (0.71)	92 (0.82)	98 (0.82)	269 (0.28)
Under the influence of medications	-	-	-	-	-	-	-	-	732 (6.57)	809 (7.24)	929 (7.77)	2470 (2.57)
Not specified/not established the agent	3733 (63.13)	3472 (63.64)	3334 (65.06)	3676 (63.48)	5974 (70.24)	6432 (60.02)	6093 (61.09)	5815 (58.96)	5575 (50.05)	5526 (49.49)	5777 (48.30)	55 407 (57.55)
Overall	5942 (6.17)	5484 (5.70)	5150 (5.35)	5824 (6.05)	8642 (8.98)	10,282 (10.68)	10,052 (10.44)	9912 (10.30)	11,358 (11.79)	11,424 (11.87)	12,201 (12.67)	96,271 (100.00)

Note: data concerning a victim may appear in several items. Source: own study based on https://statystyka.policja.pl/st/wybrane-statkieta/zamachy-samobojcze (accessed on 30 November 2020).

## Data Availability

Datasets used and analyzed during the current study are available from the corresponding author on reasonable request.
